# Stochastic Simulations of Pattern Formation in Excitable Media

**DOI:** 10.1371/journal.pone.0042508

**Published:** 2012-08-10

**Authors:** Matthias Vigelius, Bernd Meyer

**Affiliations:** FIT Centre for Research in Intelligent Systems, Monash University, Clayton, Victoria, Australia; Université de Nantes, France

## Abstract

We present a method for mesoscopic, dynamic Monte Carlo simulations of pattern formation in excitable reaction–diffusion systems. Using a two-level parallelization approach, our simulations cover the whole range of the parameter space, from the noise-dominated low-particle number regime to the quasi-deterministic high-particle number limit. Three qualitatively different case studies are performed that stand exemplary for the wide variety of excitable systems. We present mesoscopic stochastic simulations of the Gray-Scott model, of a simplified model for intracellular Ca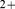 oscillations and, for the first time, of the Oregonator model. We achieve simulations with up to 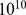 particles. The software and the model files are freely available and researchers can use the models to reproduce our results or adapt and refine them for further exploration.

## Introduction

Excitability is a common trait found in numerous complex systems arising in areas as diverse as physical chemistry [1], [2], neuroscience [3], and cell physiology [4], [5]. Excitable media are typically governed by nonlinear dynamics and characteristically exhibit a rest state, an excited state and a refractory period [6]. Diffusive excitable systems can display a wide variety of intricate patterns with a high degree of spatial organization, such as target patterns, spiral waves, and three-dimensional scroll rings [5], [6]. In most applications, the systems in question are subject to a considerable amount of noise such as experimental (external) noise or internal fluctuations due to the low numbers of particles involved. The presence of noise can qualitatively change the system characteristics and can lead to new and previously unobserved effects [7], [8]. For these reasons, a comprehensive picture can only be obtained if noise is included in the model and can be correctly simulated.

Realistic systems are, owing to their complexity, typically not accessible to an analytic approach and researchers must resort to computationally expensive stochastic simulations. The limitations posed by the available resources force users to carry out simulations in either of the two limiting regimes: the low-noise approximation, where an essentially deterministic, macroscopic description is amended by a suitable noise term [7]–[9], and the noise-dominated microscopic regime which involves dynamic Monte-Carlo simulations to track the time evolution of individual particles on a microscopic level [10]–[15]. The (internal) noise-dominated regime occurs when the particle density is low while the deterministic low-noise approximation corresponds to a high particle density.

The chief contribution of the research presented here is to demonstrate how large-scale Monte-Carlo simulations of active media are now at a point where they can capture emergent macroscopic behavior which is traditionally modeled using macroscopic reaction-diffusion equations. Due to the high number of particles involved, the macroscopic limit was previously unaccessible to particle-based stochastic algorithms. Recently, the authors developed new high-performance solvers [16], [17] that allow us to venture into this regime. This is significant for two reasons. Firstly, emergent effects such as pattern formation typically only occur when a sufficient amount of individual entities are present. For example, two of our models, the Gray-Scott system and the Oregonator model, require a macroscopic number of particles to exhibit spiral waves. On the other hand, coherent intracellular Calcium waves can already form at small particle numbers. Hence, if one is interested in exploring the conditions for emergent behavior, it is mandatory to use a unified approach that covers the low and high particle number regime on equal grounds instead of switching between different approximations. Secondly, many problems, for example stochastic models of the intracellular Calcium distribution, involve particle counts on vastly different scales. The standard approach to simulate these systems is to couple stochastic models for receptor dynamics with a quasi-deterministic description of the Calcium ions [5]. In this article, we will demonstrate how it is now possible to use a unified approach based on dynamic Monte-Carlo simulations to capture the dynamics of intracellular Calcium waves.

### Pattern Formation under the Influence of Noise

In this subsection, we briefly summarize previous work that is dedicated to study pattern formation under the influence of noise. This concise summary will serve to motivate our case studies. Namely, we present three example systems in this article. First, we implement the Gray-Scott model [1], which, despite its attractive simplicity, produces a wealth of distinct patterns [18]. Moreover, it has been recently modeled using a stochastic approach [12] and it can hence serve as a test model for our implementation. Second, we simulate the Oregonator model for the chemical Belousov-Zhabotinsky (BZ) reaction [2]. The BZ reaction is easily accessible in a laboratory setting and provides an important model system to experimentally study pattern formation in chemical systems. Finally, we implement a model for intracellular 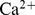 waves. Calcium waves are, without doubt, of immense biological relevance [5] and it is widely accepted that a stochastic approach is required to capture the full dynamics [19].

It is now widely recognized that noise plays an important part in the formation of patterns in excitable media [7]. While an extensive body of literature is dedicated to the deterministic properties of active media [5], [6], [20], the literature concerned with the influence of noise is extensive and fragmented. We refer the reader to the comprehensive reviews that exist in that area [7], [8]. The greater part of the research focuses on the constructive effect of externally applied noise and a variety of rather counterintuitive results emerged in that context. Most notably, random fluctuations can push a system from sub-excitable into the excitable regime [21]–[24] or from excitable to oscillatory [25] or back [26]. Noise can also induce pattern-formation [27] and complex spiral dynamics in excitable and sub-excitable media [28]–[31]. Of particular biological relevancy is the observation that noise alone can induce intercellular Calcium waves in diffusively coupled cells [32], [33]. These phenomena are collectively known as (spatiotemporal) stochastic coherence and are not restricted to external noise but can also be observed in models when only internal fluctuations are considered [13]. Moreover, noise can enable the formation of new patterns that are unobservable in a purely deterministic description [34]. For example, novel and previously unobserved patterns could be found in the Gray-Scott model with internal noise [9], [12], [35], [36]. Internal noise can extract characteristic frequencies in the FitzHugh-Nagumo model [13].

Recent research systematically explores the influence of internal fluctuations on Turing patterns. Stochastic simulations of the Brusselator model demonstrate that these patterns are generally robust against internal fluctuations [14]. An analytic treatment of the chemical master equation in the system-size expansion reveals that internal stochastic seed fluctuations give rise to spatially ordered macroscopic Turing patterns [37]. This analysis can be extended to growing domains and it was shown that stochastic systems support Turing patterns beyond the deterministic Turing parameter range [38]. More recently, the effect of time-delayed reactions on the formation of stochastic Turing patterns was investigated [39].

The most natural pattern that arises in a two-dimensional excitable system is the spiral wave since any wavefront with fragmented ends will eventually curl up [10], [40]. The deterministic theory of spiral waves in systems with an N-shaped nullcline [5], [6], [40] or a 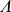-shaped nullcline [41] is well understood. Noise can support the propagation of spiral waves [28] and externally applied random perturbations can trigger complex behavior in the motion of the spiral [31], [42]. An interesting question that is relevant to medical research concerning ventricular fibrillation is the question of the stability of spiral waves [5] and spiral wave breakup [7], [43]–[46]. Spiral waves under the influence of noise were simulated using lattice gas automata [10], [47].

Typically, the analysis of pattern formation in excitable systems is carried out using the Langevin approach, where a deterministic equation is amended by a rapidly fluctuating random noise term [7]–[9]. The target quantity, for example a de-dimensionalized function for the copy count of a particular species, i.e. the number of particles contained in an (infinitesimal) subvolume, is assumed to be continuous over time and can be described by a stochastic differential equation or the equivalent Fokker-Planck equation. While this approach is valid in the macroscopic (many particle) limit, it fails when the number of particles is low. In this case, the discrete nature of the process must be taken into account and the chemical reaction is better defined as a discontinuous jump process, which is described by the chemical master equation (CME) [48], [49]. Unfortunately, directly solving the CME is computationally expensive [50]–[54]. Adding spatial effects, i.e. allowing the particles to perform (possibly biased) random walks through the domain, aggravates the problem. A mesoscopic approach compromises between computational speed and accuracy in this situation. Instead of computing spatial trajectories for each particle individually, the computational domain is divided into subvolumes and only the total number count of each particle class inside each subvolume is stored. Reactions between particles are described stochastically by the CME. Diffusion is implemented as a stochastic transition between neighboring subvolumes and can be integrated directly into the master equation [49], [55]–[60]. Recent advancements in computer hardware made it possible to numerically study the formation and evolution of structures in this fashion but these simulations are generally restricted to low particle numbers [10]–[15].

An alternative method that is particularly well-suited for an implementation on data-parallel hardware, such as graphics-processing units (GPUs), is to treat diffusion separately from chemical reactions with a stochastic-stochastic hybrid algorithm [16], [17], [61], [62]. Runtime gains of up to two orders of magnitudes are achievable with this approach [16]. In addition to parallelizing over the computational domain, one can run multiple experiments on separate GPU nodes in a cluster and hence introduce a second level of parallelization. This two-layer technique allows to perform computation-intensive tasks, such as parameter sweeps. In particular, it allows a mesoscopic approach to simulate particle numbers that can normally only be treated in a macroscopic framework.

The focus of this article is on using the mesoscopic approach described above to stochastically model the whole spectrum between the low particle number regime and the deterministic limit (high number of particles) for spatially extended excitable systems with a single algorithm. As motivated above, we concentrate on three example systems, namely the Gray-Scott model, the Oregonator reaction system and a model for intracellular Calcium dynamics in the stochastic limit. Notably, to the best of our knowledge pattern formation in the BZ reaction has not been treated from first principles in a mesoscopic fashion before. The research presented here does not seek to shed new light on the conditions and properties of excitability in these systems. Rather, the purpose of this article is to demonstrate that state-of-the-art numerical modeling makes mesoscopic simulations of complex chemical reaction systems comparably effortless and easily allows previously unattainable applications such as exploration of the parameter space. We present the first such simulations of pattern formation in excitable media in the very high density regime. As a side effect, we show how the particle density influences the formation of distinct patterns.

### Article Structure

The article is structured as follows. In the Methods section, we briefly describe our implementation of a hybrid stochastic simulation algorithm. The experiments presented here were made possible by our large-scale parallelization approach which we introduce in this section as well. We also briefly touch upon the deterministic limit of a stochastic description. The results of our numerical experiments are discussed in the Results section. We perform experiments for three different excitable systems, namely the Gray-Scott model, the Oregonator model and a simplified model for Calcium oscillations. All three models are described here. The article finishes with a discussion of the results and an outlook on further research opportunities.

## Methods

### Stochastic-stochastic Hybrid Simulation Algorithm

The algorithm underlying our implementation has been elaborated elsewhere [16], [17]. Here, we briefly recap the main points for the convenience of the reader.

As in any mesoscopic algorithm, we divide the computational domain into cubic subvolumes with equal side length 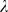. Each subvolume is assumed to be perfectly stirred such that the particle concentration of each species is homogeneous *inside* the subvolume but can vary *between* subvolumes. In this discretization, diffusion is modeled as a jump process between neighboring cells with a jump probability that depends on the local diffusivity and drift. In the same manner, chemical reactions are discrete transitions between states.

**Table 1:** Simulation runtimes of the Gray-Scott model.

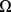	Runtime in seconds
(deterministic)	2814
2.5	58
25	85
250	301
25000	21673

Simulation runtime for the Gray-Scott model Eqs. (1)–(4).

**Table 2:** Simulation runtimes for the Oregonator model.

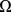	Runtime in seconds
(deterministic)	638
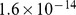	67
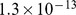	241
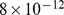	11511
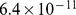	90874

Simulation runtimes for the Oregonator model of the BZ reaction Eqs. (7)–(14).

**Table 3:** Simulation runtimes for the 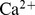 model.

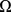	Runtime in seconds
(deterministic)	454
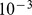	15
0.125	71
1	595
125	71299

Simulation runtimes for the 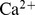 model Eqs. (18)–(26).

Both processes, reaction and diffusion, can be combined in a coupled chemical and diffusion master equation [49]. Solving this combined master equation by standard stochastic algorithms is possible and several popular software packages choose this approach [55]–[59]. However, it is clear that a fine granularity of the computational grid and a large number of particles greatly increase the frequency of transitions and hence the computational cost. This inherent difficulty is compounded by the fact that most standard algorithms cannot be easily parallelized [55], [63] and, consequently, a large part of the portion of the relevant parameter space is inaccessible to this approach.

A potential remedy to this problem can be found by treating reactions inside subvolumes separately from diffusion between subvolumes in a so-called *stochastic-stochastic hybrid* algorithm [61], [62]. As we will demonstrate in the next section, this technique, which is also known as *operator splitting*, is well suited for a data-parallel implementation [16], [17]. One example for the stochastic-stochastic hybrid algorithm is the Gillespie Multiparticle (GMP) method [61], [64], which employs a common time step over the whole domain. The global time step allows us to advance all subvolumes synchronously in time during one simulation step without the need for asynchronous communication between subvolumes. The simulation step first performs reactions between species individually *in each cell* with the standard Gillespie algorithm [63]. The particles are then propagated *between* cells according to transition probabilities that are chosen to reflect the local diffusivity and drift field [17], [64]. We finish the loop by computing the new global time step.

**Table 4:** Simulation parameters for the Gray-Scott model.

Parameter
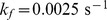
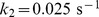
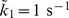
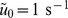
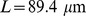
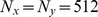
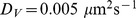

Simulation parameters for the Gray-Scott model Eqs. (1)–(4).

**Figure 1:**
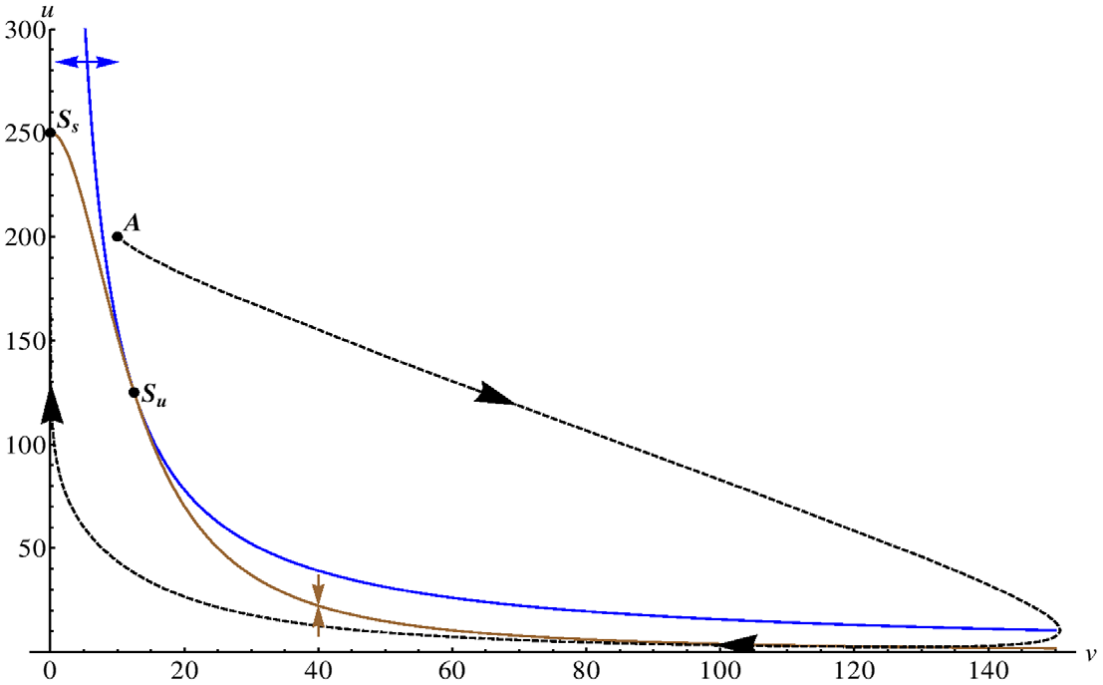
Nullclines in the Gray-Scott model. We display the nullclines of the activator species 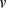 (blue curve) and the inhibitor 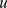 (brown curve) for the Gray-Scott model (without diffusion) in the deterministic limit for the parameter set given in Table 4. The blue (brown) arrow illustrates the direction of the gradient in phase space of the activator (inhibitor) on either side of the nullcline and the unstable fix point is marked with 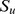. We demonstrate that the system is in the excitable regime by plotting an example trajectory (dashed curve) for a larger perturbation, starting at point 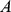, from the stable homogeneous state (marked by 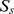 in the figure). The system relaxes towards 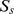 via a long excursion.

### Graphics-card Acceleration

In order to maximize both, accessibility to a broad audience and simulation performance, we implement a parallel version of GMP on graphics-processing units (GPUs). Most common workstations have built-in GPUs. In addition, designated high-end GPU arrays can be used to optimize performance. This strategy provides additional benefits by making high-performance computing accessible to researchers without access to designated computing clusters. On the flip side, the specialized hardware design and the corresponding programming model strongly limits the field of application. GPUs perform best when a multitude of threads execute the same set of instructions on different data, a programming model commonly termed data-parallel. If, however, divergence between threads is high, i.e. different threads perform different instructions due to differently evaluated conditionals in the code, the speed benefit is quickly lost and the overall performance drops.

**Figure 2:**
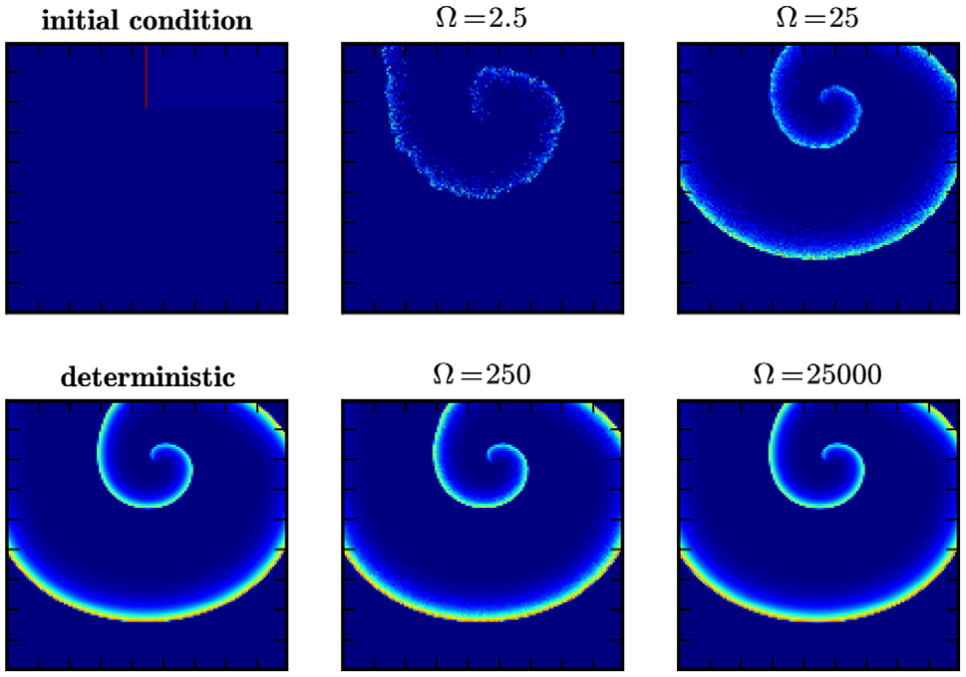
Formation of a spike spiral wave in the Gray-Scott model. Shown are snap-shots of a spike spiral wave in the Gray-Scott model Eqs. (1)–(4), initialized as shown in the top left panel, at 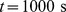 in the deterministic simulation (bottom left) and in stochastic simulations for different scale factors 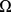 (rightmost columns).

We can easily achieve a data-parallel implementation of GMP by assigning each subvolume of the computational domain to an individual thread on the device. The Gillespie algorithm treating the reactions can then be performed independently by each thread for the duration of the time step. Diffusion is then completed at the end of the time step and the host is responsible for global synchronization between time steps. Details about our GPU implementation are provided elsewhere [16], [17].

### Large-scale Parallelization

Two requirements pose significant difficulties in the context of stochastic simulations. Firstly, many applications require a large number of experiments to achieve statistical validity. Secondly, a systematic exploration of the parameter space, including more sophisticated applications like parameter optimization, demand a sufficient amount of sample points. We demonstrate how these obstacles can be overcome by introducing a second level of parallelization over different experiments.

**Table 5:** Identification of symbolic species in the Oregonator model.

A	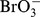
B	oxidizable organic species
X	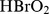
Y	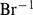
Z	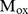
R	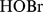

Identification of the species in the FKN representation (after [6]).

The key strategy here is to bundle several GPU-enabled computing nodes into a GPU cluster. We run our simulations on the Monash Sun Grid (http://www.monash.edu.au/eresearch/services/mcg/msg.html) which currently comprises five nVidia Tesla S1070 quad-GPU arrays. The burden of managing the resources and, in particular, evenly balancing the computing load between nodes is carried by the middle-ware Nimrod (http://www.messagelab.monash.edu.au/Nimrod). Our web-based front end, Inchman (http://www.csse.monash.edu.au/~berndm/inchman/), allows researchers to define their reaction-diffusion model and the required simulation task on the web interface and submit the project to our GPU cluster for processing. All models used in this article can be found in the public repository on the web site.

**Table 6:** Simulation parameters for the Oregonator model.

Parameter
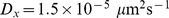
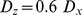
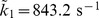
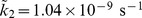
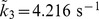
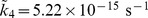
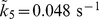
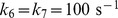
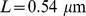
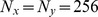

Simulation parameters for the Oregonator model of the BZ reaction Eqs. (7)–(14).

### From Micro to Macro: the Deterministic Limit

We are mainly interested to model the whole spectrum from the low-particle, i.e. fluctuation-dominated, to the high-particle, quasi-deterministic, end. Mathematically, it is possible to expand the multivariate reaction-diffusion master equation in terms of a parameter 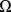 such that the deterministic equations are recovered in the limit 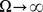 [49], [65]. We will perform simulations of all models over a broad range of 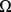. As a main result, we will demonstrate below how systems can undergo qualitative changes for different values of 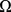. Our technique enables us to model this transition with a unified approach. The expansion, called *system size expansion*, allows considerable freedom in choosing which system property is used as the expansion parameter 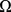. Here, we decide to scale the concentrations of the chemical compounds, i.e. 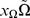, while leaving the subvolume size constant [12]. Reaction dynamics is preserved as long as, at the same time, we scale the rate constants 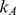 for a particular reaction 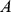 according to 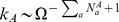, where 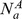 denotes the number of particles of species 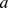 which take part in the reaction 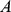 [49]. Naturally, the initial concentrations need to be scaled correctly with 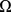 as well.

**Figure 3:**
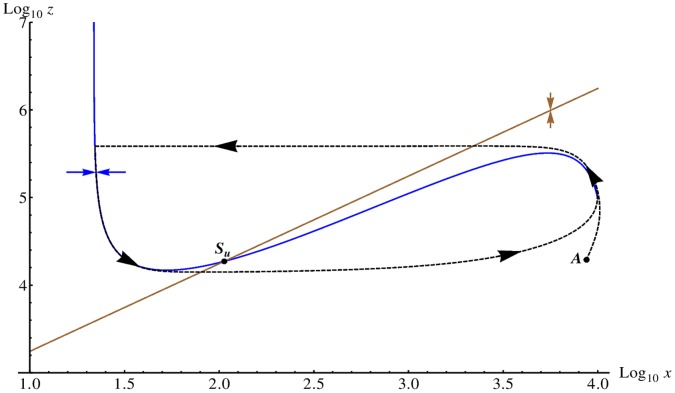
Nullclines in the Oregonator model. We display the nullclines (logarithmic scale) of the activator species 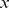 (blue curve) and the inhibitor 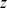 (brown curve) for the Oregonator model in the deterministic limit for the parameter set given in Table 6 and 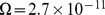. We assume that the intermediary species 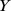 is in a steady-state equilibrium with 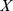 and 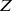 and ignore diffusion. The blue (brown) arrow illustrates the gradient in phase space of the activator (inhibitor) on either side of the nullcline and the unstable fix point is marked with 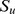. The system is in the unstable (oscillatory) regime. We plot an example trajectory (dashed curve) of a larger perturbation from the (linearly stable) trivial homogeneous state. Starting at point 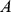, the system enters a limit cycle in phase space.

We compare our stochastic simulations with a direct solution of the corresponding deterministic partial differential equation. The deterministic solver we use is, similarly to the stochastic solver, based on an operator splitting approach where reactions are decoupled from the diffusion operator. The reaction network is solved for the diffusion time-step with a semi-implicit solver based on a steady-state approximation (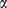-QSS), which is optimized for solving stiff differential equations [66]. The Laplace-operator for diffusion is discretized with a second-order accurate, centered difference scheme. For optimal performance, we implement a GPU-accelerated, data-parallel version of the solver and integrate it into the Inchman framework. Details of the implementation will be provided elsewhere.

**Figure 4:**
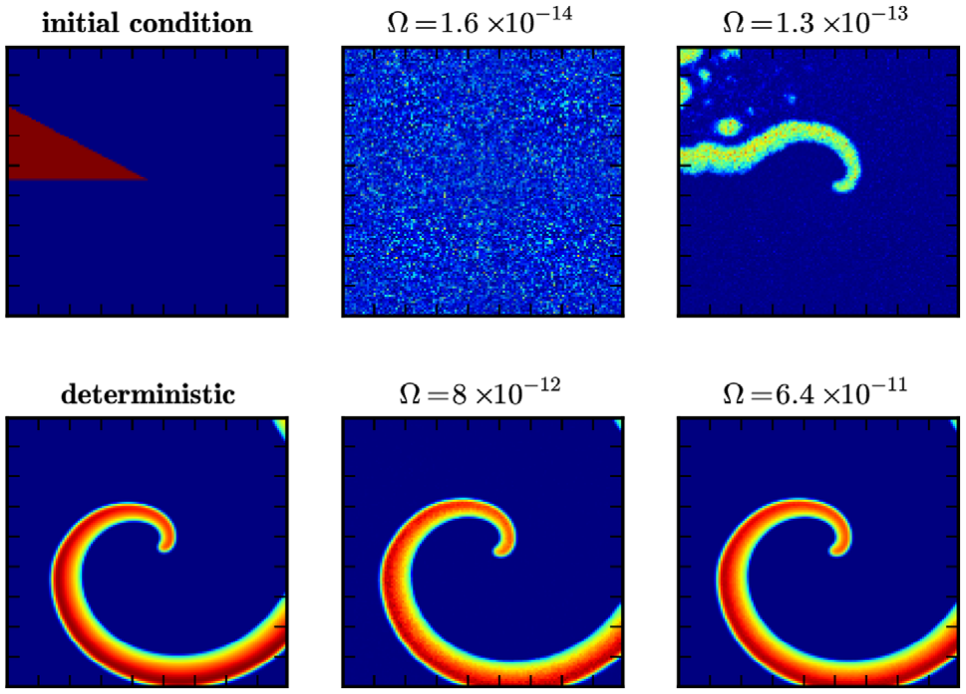
Formation of a spiral wave in the Oregonator model. Shown are snap-shots of the formation of a spiral wave in the Oregonator model for the BZ reaction Eqs. (7)–(14), initialized as shown in the top left panel, at 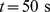 in the deterministic simulation (bottom left) and in stochastic simulations for different scale factors 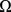 (rightmost columns).

### Simulation Setup

All simulations were performed on the GPU cluster of the Monash e-Research Centre (http://www.monash.edu.au/eresearch/). The cluster consists of currently ten nodes equipped with NVIDIA Tesla S1070 quad-GPU arrays, allowing us to benefit from the large-scale parallelization approach. We emphasize that all individual runs were also tested on a standard work station (INTEL E6550 dual core CPU at 2.33 GHz with 2GB RAM and an NVIDIA Quadro 2000 GPU card) which was found to perform equally well with a comparable runtime.

The overall runtime for individual simulations varies from a couple of seconds to about 25 hours. Tables 1, 2, and 3 give the runtimes for the Gray-Scott model, the Oregonator scheme, and the Calcium-wave simulations, respectively.

**Figure 5:**
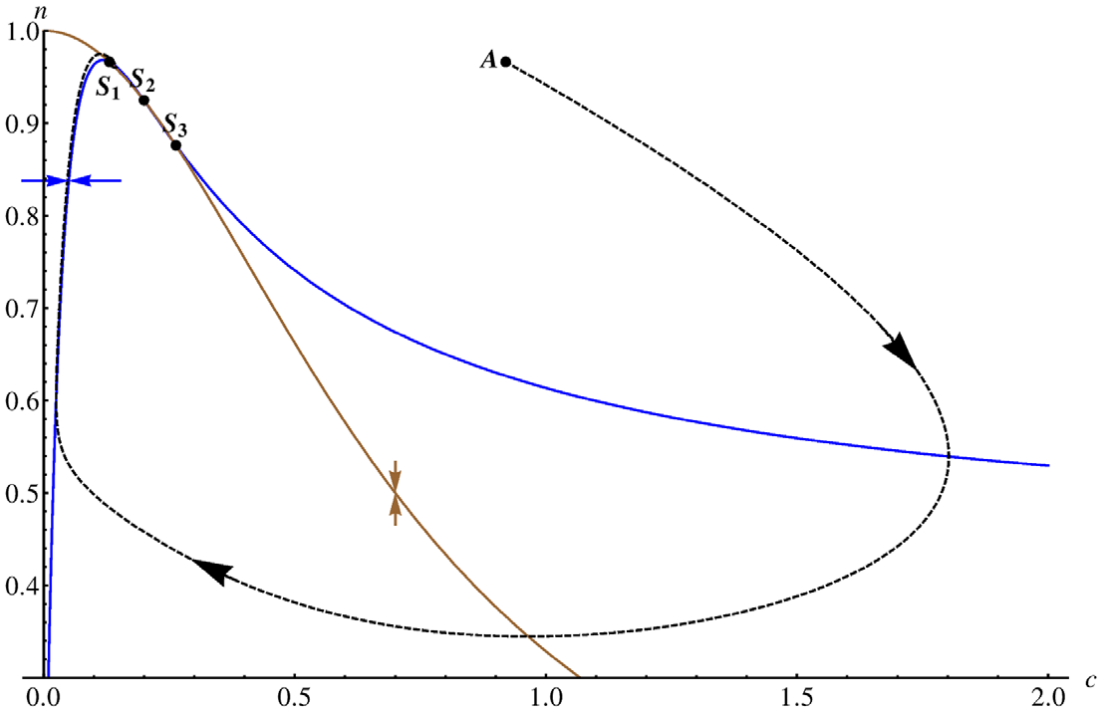
Nullclines in the 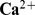 model. We display the nullclines of the 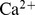 concentration 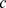 (blue curve), which can be regarded as the activator, and the fraction of open channels 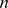 (brown curve) for the Calcium oscillation model in the deterministic limit [Eqs. (27)–(29)] for the parameter set given in Table 7 and assume that diffusion is switched off. The blue (brown) arrow illustrates the gradient in phase space of the activator (inhibitor) on either side of the nullcline and the fix points are marked with 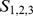. We plot an example trajectory (dashed curve) of a larger perturbation from the homogeneous state 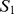. Starting at point 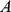, the system relaxes towards 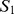 via a long excursion.

**Table 7:** Simulation parameters for the 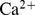 model.

Parameter
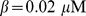
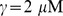
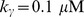
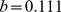
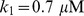
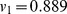
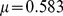
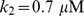
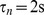
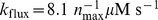
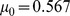
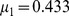
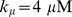
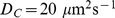
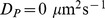
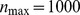
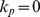
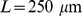
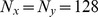

Simulation parameters for the 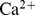 model Eqs. (18)–(26). With the length 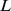 and number of grid cells 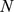 given, we find for the subvolume size 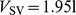 and can therefore convert the concentration base 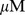 into the number of particles per subvolume 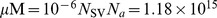.

We prescribe “reflecting” boundary conditions for all stochastic simulations, i.e. no particles are allowed to leave the domain. This is implemented by rejection sampling: if a particle tries to leave the integration domain through this boundary, it “bounces” back and stays in its subvolume [16], [17].

## Results

In this section we discuss our three case studies. The example systems in these case studies display qualitatively different behavior and, as a whole, cover a broad spectrum of commonly observed effects in excitable media. The structure of the according subsections is similar for each system. We start by briefly introducing the system in question and discuss details of the excitability properties in the deterministic limit. We then set up our stochastic implementation of the model and present the results of the simulation focusing on if and how spiral waves form when the particle number, which scales with the expansion factor 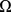, is changed. We start our discussion with the Gray-Scott model in the next subsection. We then turn to the Oregonator system and conclude with a simplified model of intracellular 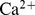 oscillations. We stress again that the main intention of this article is to demonstrate how our approach is capable of simulating a wide range of particle numbers and cover the whole regime from fluctuation-dominated (small particle count) to quasi-deterministic (large number of particles). The models are presented in order of increasing complexity. To the best of our knowledge, only the Gray-Scott system has been treated with a stochastic simulation approach before [12].

### Gray-Scott Model

The Gray-Scott model was devised in an attempt to systematically investigate complex isothermal autocatalytic reactions and provides a simple prototype for these systems [1]. Using this comparably simple system, we demonstrate how macroscopic features emerge from a microscopic description when 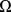 is increased. In particular, we will see that a macroscopic amount of particles is required to permit the formation of coherent spiral waves. The Gray-Scott system has been previously modelled in this regime using stochastic simulations [12].

The model studied here is comprised of a species 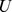, the inhibitor, that reacts in a cubic autocatalytic step with a second species 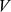, the activator. We assume a finite life time of both species and, in addition, allow constant inflow of species 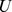 into the system. The chemical reaction scheme can then be written as


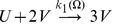
(1)


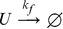
(2)


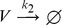
(3)


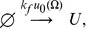
(4)

where we remind the reader that 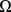 scales the reaction rates as 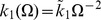 and 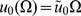. Remember that first-order reactions need not be scaled. We furthermore introduce the control parameters 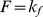 and 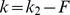 such that the properties of our system are fully determined by its position in the 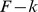 plane [1], [12], [18]. Following standard modeling procedures for Gray-Scott systems [12], we allow the activator species 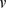 to diffuse freely, with diffusivity 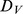, while the inhibitor species is assumed to be spatially pinned. The system then exhibits a wealth of different patterns [9], [18].

In the deterministic limit (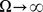), we can write down the evolution of the averaged copy counts per subvolume of each species as a set of partial differential equations,


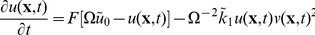
(5)

and


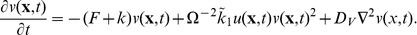
(6)

Note that, in Eqs. (5) and (6), 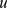 and 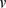 denote the actual *mean copy count* of species 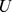 and 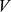, viz. the number of particles in each infinitesimal subvolume averaged over many experiments. Consequently, 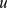, 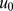 and 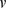 are dimensionless while the reaction rates 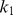, 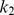 and 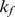, and hence 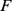 and 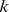, have dimension 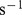. Naturally, 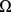 is dimensionless as well and 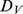 has the usual dimensions of a diffusivity, 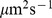. We choose this representation to allow easy comparison with the simulation results, which are given as number of particles per subvolume. Converting Eqs. (5)–(6) into concentrations is straight forward.

We plot the nullclines of Eqs. (5)–(6) for the parameter set given in Table 4 (Fig. 1). Shown are the nullclines for the activator (brown curve) and inhibitor (blue curve). The nullcline of the activator evolution equation is 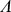-shaped and, depending on the value of the dimensionless bifurcation parameter 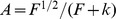, allows either one (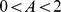), two (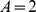) or three 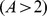 homogeneous stationary states [67]. It is possible to excite ultrafast traveling spike auto-solitons that allow the formation of two-dimensional spike spiral waves [41]. For our simulations, we choose a parameter set such that the system is located on the saddle-node bifurcation curve (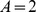) [67]. For this parameter set, we have two homogeneous fix points, 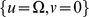 (labeled 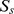 in the figure) and 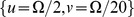 (labeled 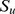), which are linearly stable and unstable, respectively. The system is clearly excitable as the trajectory of a typical large perturbation in phase space (dashed curve in Fig. 1 ) demonstrates. Starting at point 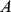, the system is forced to relax via a long excursion. As soon as it passes the brown curve, the system is in the refractory regime where further perturbations have no effect.

We set up our simulations with the parameters given in Table 4. The integration domain is a square, with side length 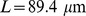, that is divided into a grid of 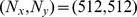 subvolumes. Only species 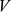 is allowed to diffuse with 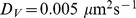. At 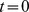, the integration domain is set to the (stable) homogeneous state 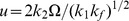 and 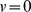. We initiate the formation of a spiral wave by applying a perturbation from the homogeneous background, 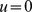 and 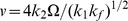, to a small rectangular region 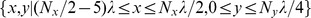. The symmetry breaking of the wave is induced by initializing the right half of the domain, 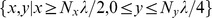, in the refractory regime 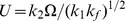 and 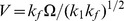.

The results are presented in Fig. 2. We display the particle count per subvolume of the activator species 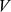 at simulation time 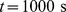. The top-left panel shows the initial configuration of the experiment and the right-most columns illustrate the effect of the scaling parameter 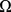 in the stochastic simulation. For comparison, we also include the corresponding snap-shot of the deterministic model (bottom left). We observe a distinct spiral pattern for 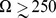, corresponding to 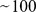 particles per subvolume. In the high-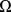 regime, 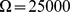, the result is virtually indistinguishable from the deterministic experiment. This example illustrates neatly how the system-size expansion indeed reproduces the deterministic limit. Note that the case 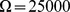 corresponds to about 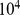 particles *per subvolume*, which translates to a total of 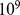 particles in the integration domain. This regime would be inaccessible without the GPU-accelerated implementation we employ here.

As expected, nucleation of the spiral wave breaks down in the microscopic regime. For 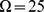 (top middle panel), the wave-front quickly dissipates and the inner part of the spiral wave is only faintly visible. In the low-number limit (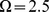, corresponding to about 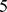 particles per subvolume) we do not observe any spiral structure. While the interface between the stable homogeneous state and the refractory region is still distinct, the area behind the passing wave front is nearly homogeneous. In particular, we find no evidence for nucleation of thermal waves, which are noise-sustained wave patterns in subexcitable media [12], [28], [68]. In summary, this subsection demonstrates nicely how our method allows to cover a wide parameter range with genuinely stochastic simulations.

### Oregonator Model

We now turn our attention to the celebrated Belousov-Zhabotinsky (BZ) reaction family [69], [70]. It is widely regarded as the archetype of an oscillating chemical reaction and, most generally, involves oxidation of an organic species by bromic acid, catalyzed by metal ions. Its particular appeal to researchers stems from the fact that it can be easily reproduced in a laboratory setting and the system has been extensively studied [71]. In fact, various recipes exist to perform the experiment at home [6].

The ease of handling makes the BZ reaction an ideal test bed to study the influence of external noise on the formation and dynamics of patterns. Experimentally, a light-sensitive catalyst can be used to control the excitability of the reaction with a high spatial and temporal resolution [8], [72]–[74]. Using this technique, sophisticated experiments have been performed to elucidate the dynamics of spiral waves in the BZ reaction [25], [42], [75], [76].

The oscillatory properties of the BZ reaction can be understood in terms of the Field-Körös-Noyes (FKN) mechanism [6], [77]. While the details of this model are fairly complex and of little interest here, it is worth mentioning that 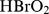 plays the role of the activator in an autocatalytic reaction that is inhibited by bromide ion. A considerable simplification is provided by the popular Oregonator model [78]. The Oregonator reaction scheme is an abstract representation of the FKN mechanism, consisting of six reactants that can be identified with the chemical compounds in the BZ reaction (we give the identification in Table 5), and five reactions, namely


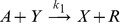
(7)


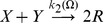
(8)


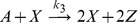
(9)


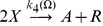
(10)


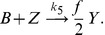
(11)

Here 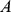 and 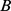 are assumed to be constant and we subsume them into the reaction constants such that reaction (7) is replaced by the unimolecular reaction 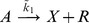 where 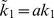 (with 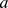 the constant copy count per subvolume of 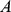). Reactions (9) and (11) are rewritten accordingly. The reaction rates 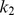 and 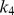 scale as 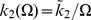 and 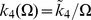. Essentially, 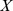 is the activator and 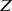 the inhibitor. 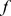 is a bifurcation parameter and it can be shown that the steady state of system (7)–(11) is unstable for 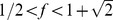 [6]. In terms of the taxonomy for active media, the Oregonator model is characterized by the upside-down N-shape of the activator nullcline.

Numerous numerical studies about the formation and dynamics of patterns in diffusive Oregonator systems exist [2], [79], [80], some of them dedicated to the influence of noise [8], [25], [81]. However, to the best of our knowledge, a mesoscopic, genuinely stochastic simulation of the three-state Oregonator system as given by Eqs. (7)–(11) has not yet been achieved. The multi-scale parallelization model provided by Inchman allows us to present such an experiment here.

The aim is to stochastically reproduce spiral waves as observed in deterministic simulations [2]. To this end, we allow diffusive motion of the activator species 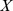 and the inhibitor species 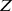, while 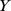 remains spatially frozen. Our simulations are carried out in the unstable (oscillatory) regime 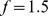 [2]. Reaction (11) then assumes a fractional stoichiometry. While this makes perfect sense as a mathematical abstraction to encapsulate intermediate reactions and products, it cannot be modelled in a discrete domain. We therefore introduce two artificial species, 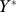 and 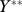 and replace Eq. (11) with three helper reactions


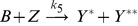
(12)


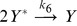
(13)


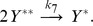
(14)

Provided the time-scales of reactions (13) and (14) are much smaller than any other time-scales involved, the chemical dynamics are correctly reproduced. Note that it is not necessary to scale 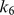 and 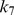 provided they are larger than any other reaction rates.

We can write deterministic equations for the evolution of the copy counts as a set of three coupled partial differential equations,


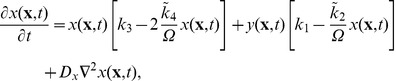
(15)


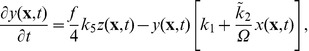
(16)

and


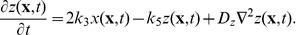
(17)

We chose a parameter set, given in Table 6, that is designed to recover literature results of deterministic simulations of the two-state Oregonator model in the limit 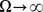 [2]. As in the previous subsection, the simulation domain is a square region (side length 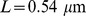) divided into subvolumes by a 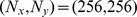 regular lattice with spacing 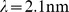. A spiral wave is induced following the recipe of Jahnke *et al.* [2]. We set species 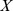 to the stationary equilibrium value 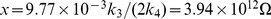 everywhere except for a narrow wedge (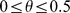 where 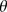 denotes the polar angle) where we set an overdensity (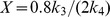). The inhibitor species 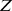 is initialized according to the formula 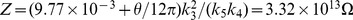. Finally, we initialize 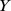 to the dynamic equilibrium value, viz. 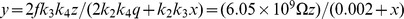.

The nullclines for the time evolution of 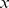 and 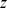 from Eqs. (15) and (17) are plotted in Fig. 3, where we switch off diffusion and also assume that species 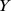 is in steady-state equilibrium with 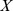 and 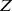 [6]. A typical excursion is included (dashed curve) for a perturbation starting at point 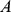 that quickly enters the limiting cycle and the system exhibits oscillations.

Fig. 4 presents the outcome of these simulations. We display the number of particles of species 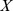 per subvolume at 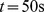 for different values of the scale parameter 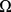. The top-left panel illustrates the wedge that is used to induce the spiral wave through an overdensity. For comparison, we also include the result of a deterministic run (bottom-left panel). The transition from the low 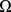 to high 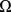 regime is clearly visible. At 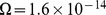 (top-middle panel) the particle number per subvolume is too low (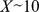 and 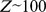) to allow coherent structure formation. We can see how a spiral wave starts to nucleate at 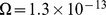 (top-right panel), corresponding to 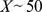 and 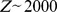, but is quickly dispersed through noise. However, the passing wave triggers target patterns in the medium (compare top-left part of the picture). A coherent spiral wave is first visible at 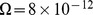 (bottom center) and for high 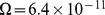 (bottom right) the pattern is virtually indistinguishable from its deterministic counterpart. We note that the high-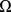 simulation contains about 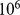 particles per subvolume. This has not been achieved before at comparable densities.

### Ca^2+^ Waves

The universal role of intracellular Calcium as a second messenger in cell physiology has been extensively investigated [5]. Numerous experiments pay special attention to the spatiotemporal behavior of the cytoplasmic 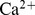 concentration following an initial agonist stimulation [4], [5], [82], [83]. These studies provide evidence for an intimate connection of intracellular Calcium to the theory of excitable media [4]. Elaborate imaging techniques reveal the existence of highly intricate patterns such as target patterns and spiral waves [5], [84]–[86]. Following the ongoing motif of this article we focus on the generation and dynamics of spiral waves in *Xenopus* oocytes [84], [86]. *Xenopus* is an African aquatic frog. Its oocytes can have a diameter larger than 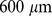 which greatly facilitates observation of macroscopic wave patterns [5].

The dynamics of cytoplasmic 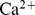 are determined by a variety of influx and pump processes depending on the cell type in question. Details about these processes can be found in standard cell physiology textbooks [5]. Here, we briefly describe the parts relevant to our simulations. The concentration gradient between cytosolic and extracellular Calcium is maintained by two separate pathways. (i) An ATPase pump can remove intracellular 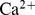 through the plasma membrane. (ii) Calcium can be stored into membrane-bound internal reservoirs, *inter alia* the endoplasmic reticulum (ER). Removal from cytosolic 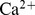 into internal storages is accomplished by a SERCA ATPase pump. Similarly, Calcium can be released from the ER into the cell through inositol (1,4,5)- trisphosphate (IP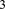) sensitive receptors. IP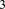, which can diffuse freely inside the cell, is a second messenger that is released following a triggering event through an external agonist stimulation. Most importantly, IP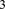 receptors are sensitive to the 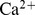 concentration which activates and inhibits Calcium release on different time scales. Finally, leakage of extracellular Calcium across the plasma membrane increases the cytosolic Calcium concentration.

By now, the importance of a stochastic approach to modelling intracellular 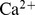 is widely recognized [5], [19]. Numerous experiments give clear evidence that 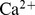 release from IP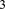 receptor (IPR) clusters occurs through a series of stochastic events called “puffs” [87].

The standard approach to capture the observed stochastic behavior is to model all diffusive processes (of Calcium and IP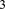) deterministically and include the release of 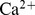 through IPRs as a Markov process with discrete events [88]–[90]. An early attempt to treat Calcium diffusion stochastically through a master equation approach was, due to limited computational resources, restricted to one spatial dimension [11]. Using a spatially homogeneous model of intracellular Calcium oscillations, Kummer *et al.* investigate the transition from the stochastic to the deterministic regime [91]. However, we are not aware of any models that are spatially resolved in multiple dimensions and treat all components stochastically.

Developing such a model is a formidable task that we do not attempt here. Instead, we implement a stochastic version of a minimal model that was successfully used to reproduce spiral wave patterns observed in *Xenopus* oocytes by Atri *et al.* [85]. The aim here is to demonstrate that, in principle, the complexity is manageable and we can perform stochastic simulations of 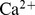 spiral waves over the whole range of particle numbers up to the deterministic (large particle number) regime. As a *single pool* model, we only keep track of the cytosolic Calcium concentration 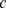 and do not separately account for 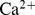 bound in the ER storage. We ignore the spatial structure of IPRs, which tend to aggregate in clusters [5], [87], [90], and instead simply assign a number of open IPRs, 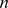, to each subvolume. 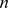 changes in response to 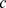 and the local IP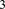 concentration, 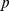. We allow 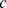 and 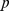 to diffuse freely inside the cell, with respective diffusion coefficients 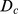 and 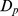. We completely ignore the capability of 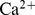 ions to bind to large proteins, a process called buffering [92]–[94].

The three constituents of the Atri *et al.* model, 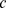, 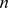, and 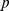, correspond to the species 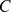, 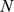, and 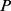. Leakage of 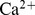 through the plasma membrane is modelled as a zeroth-order reaction:


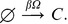
(18)

Sequestering of Calcium into the ER is implemented as a decay reaction,


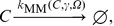
(19)

where the SERCA pump process is represented by a Michaelis-Menten type rate law


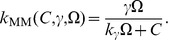
(20)

and 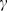 and 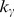 are free parameters. In writing down Eq. (20) as a rate law we neglect the possibility of pump reversal [5], [95]. Moreover, a complete stochastic approach requires modeling the underlying transport reactions on a molecular level. However, the literature model we are trying to reproduce [85] does not account for pump reversal and we leave a more elaborate model to future work.

Release of Calcium from the ER into the cell is controlled through IP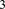-sensitive gates. As a reaction, we write


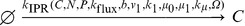
(21)

and encode the details of the channel behavior in the rate law,


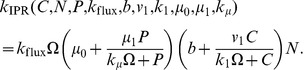
(22)

Eq. (22) is based on the assumption that each IPR has three Calcium binding sites, a single site, a domain where two 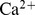 ions can bind cooperatively, and one site reserved for IP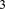. The channel is open if the single Calcium site and the IP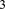 site are both active while the cooperative Calcium binding domain remains unactivated. The channel open probabilities can then be modelled with cooperative kinetics [85]. A more accurate description would be given by an eight-state IPR model, or a simplified version of it [5], [96], [97]. More recently, models with saturating binding rates were proposed [5], [98]. Implementing a full stochastic version of the eight-state IPR has been done [99] but is computationally expensive. Most simulations therefore opt for a simplified version or a Langevin approach (cf. Introduction) [5], [88]–[90].

The Atri *et al.* model of the open channel dynamics, that we adopt here, is based on the assumption that the inhibitive domain of IPR relaxes on a slower time scale 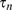 than the activating sites [85]. The activating sites reach a fast equilibrium with 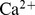 and IP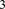 and we therefore add a creation reaction,


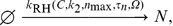
(23)

where 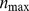 denotes the total number of IPR channels per subvolume, 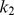 is a free parameter and the rate law is given by


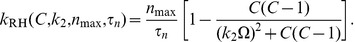
(24)

Open channels are “destroyed” on the relaxation time scale 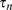 and we write


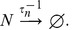
(25)

Finally, we allow breakdown of IP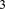 with a rate 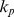:


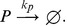
(26)

The deterministic limit of the stochastic model Eqs. (18)–(26) reproduce the partial differential equations given in Atri *et al.* [85] for the average number of Calcium ions per subvolume, 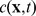,


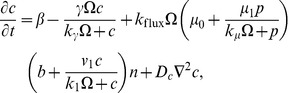
(27)

the fraction of open channels per subvolume 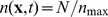,


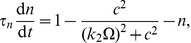
(28)

and the concentration of IP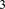, 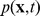,


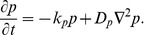
(29)

Note that we omit the argument of the dependent variables for readability. Fig. 5 displays the nullclines for 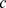 (blue curve) and 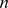 (brown curve) (with constant IP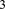 concentration). In our simplified model, we can regard Calcium ions as activator while the number of open channels acts as the inhibiting species [100]. The nullclines for this model are 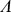-shaped and exhibit three fix points (labelled 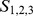). Again, we include an example trajectory (dashed curve) in the plot.

We collect the parameters we used for our simulation runs in Table 7. This particular choice corresponds to numerical models of the spiral wave formation in *Xenopus* oocytes [85]. We perform the experiments on a square integration domain with side length 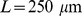 which is divided into subvolumes by a 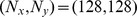 lattice, corresponding to a lattice spacing of 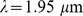. We stimulate the formation of a spiral wave, in a way analogous to the Gray-Scott model experiments, by forcing an overdensity in the Calcium concentration 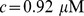 upon a rectangular region 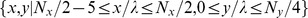 embedded in the homogeneous background 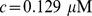, 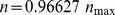, and 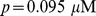. We induce breaking of the wave by initializing the right half of the domain (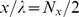) in the refractory regime 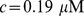 and 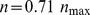.

**Figure 6:**
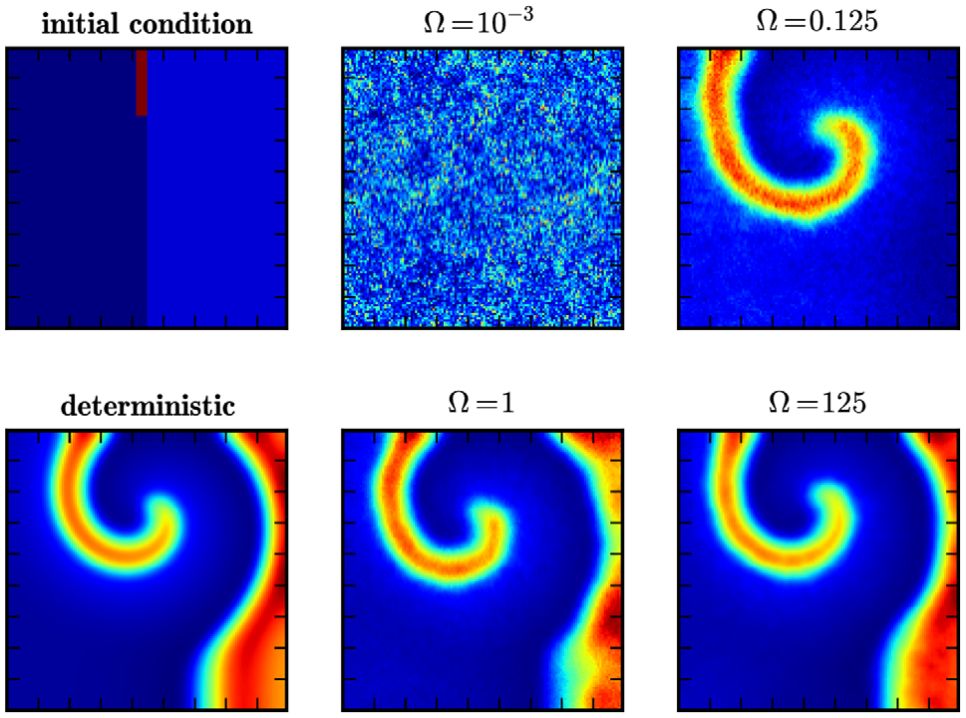
Formation of a spike spiral wave in the 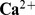 model. Shown are snap-shots of a spiral wave in the 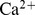 model Eqs. (18)–(26), initialized as shown in the top left panel, at 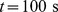 in the deterministic simulation (bottom left) and in stochastic simulations for different scale factors 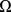 (rightmost columns).

The results of these simulations are presented in Fig. 6. Shown are the density maps (number of ions per subvolume) of 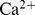 at 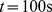 for different values of the scale parameter 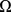 (right-most columns). The top-left panel illustrates our initial conditions and we include the outcome of a deterministic simulation (bottom-left panel) for comparison. The results concur with the findings of the other models presented above. For a small value of the scale parameter (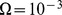 in the top-middle panel) the number count of Calcium ions per subvolume is in the order of 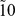 and the wave is unable to nucleate. However, even for a comparably small 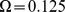 (top-right panel), corresponding to about 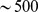 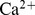 particles, the stochastic simulation approximates the deterministic results remarkably well. The spatial variance decreases for increasing 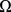. The largest of our runs, 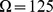 entails about 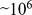 particles per subvolume. The fact that coherent waves are possible even with a small particle number underlines the importance of being able to explore the whole parameter range, from low particle numbers to high particle counts, in a single approach.

## Discussion

In this article, we demonstrate methods for stochastic mesoscopic simulations of pattern formation in excitable media. We present case studies for three qualitatively different models from chemical physics and biology which stand exemplary for the wide variety of excitable systems. Specifically, we model the Gray-Scott reaction system, as a prototype for excitability through autocatalytic reactions, the Oregonator model, which can be used to describe pattern formation in the chemical Belousov-Zhabotinsky reaction system, and finally a simplified model for intracellular Calcium waves. We introduce a two-layer parallelization approach that can be fruitfully used to achieve mesoscopic simulations with a macroscopically relevant number of particles. To the best of our knowledge, this has not been done before for the Oregonator model.

The main contribution of the research presented here is to demonstrate how efficient computation techniques allow to cover the whole range of particle counts – from the low particle regime, which is dominated by discrete fluctuations, to the deterministic high-particle number approximation – in a unified approach. This is significant. Firstly, our results numerically confirm that, as predicted by the system-size expansion, a mesoscopic jump-process description of a reaction-diffusion systems indeed approaches the correct deterministic limit as it is given by the corresponding Fokker-Planck equation. More importantly, however, a unified approach is necessary to explore how system characteristics undergo qualitative changes when the particle number is increased. The 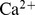 model demonstrates that it is not possible to *a priori* determine the transition point. Furthermore, if a model involves different scales of particle numbers, it is highly desirable to treat all components with a common approach. It is for these reasons that large-scale, mesoscopic simulation techniques are indispensable.

Finally, we point out that our simulation tool Inchman (http://www.csse.monash.edu.au/~berndm/inchman/) is designed as an open platform to promote collaborative research. Users can join groups and share their models and simulation results. We make the model files available in the open repositories. We invite researchers to reproduce the results presented here and encourage users to refine and adapt the models to address more sophisticated research questions. As we have demonstrated through our case studies presented here, Inchman is a valuable tool to model reaction-drift-diffusion systems in a variety of disciplines.
